# Supplementary material for: A scaffold lncRNA shapes the mitosis to meiosis switch
Source: Nat Commun. 2021 Feb 3;12:770. doi: 10.1038/s41467-021-21032-7 (PMC7859202; doi:10.1038/s41467-021-21032-7)
Supplement: Supplementary file 1 — Supplementary Information [file 41467_2021_21032_MOESM1_ESM.pdf]

Supplementary Information

A scaffold lncRNA shapes the mitosis to meiosis switch

Vedrana Andric[1,6], Alicia Nevers[1,4,6], Ditipriya Hazra[2,5,6], Sylvie Auxilien[1], Alexandra Menant[1], Marc Graille[2], Benoit Palancade[3], Mathieu Rougemaille[1,*]

Correspondence to: mathieu.rougemaille@i2bc.paris-saclay.fr

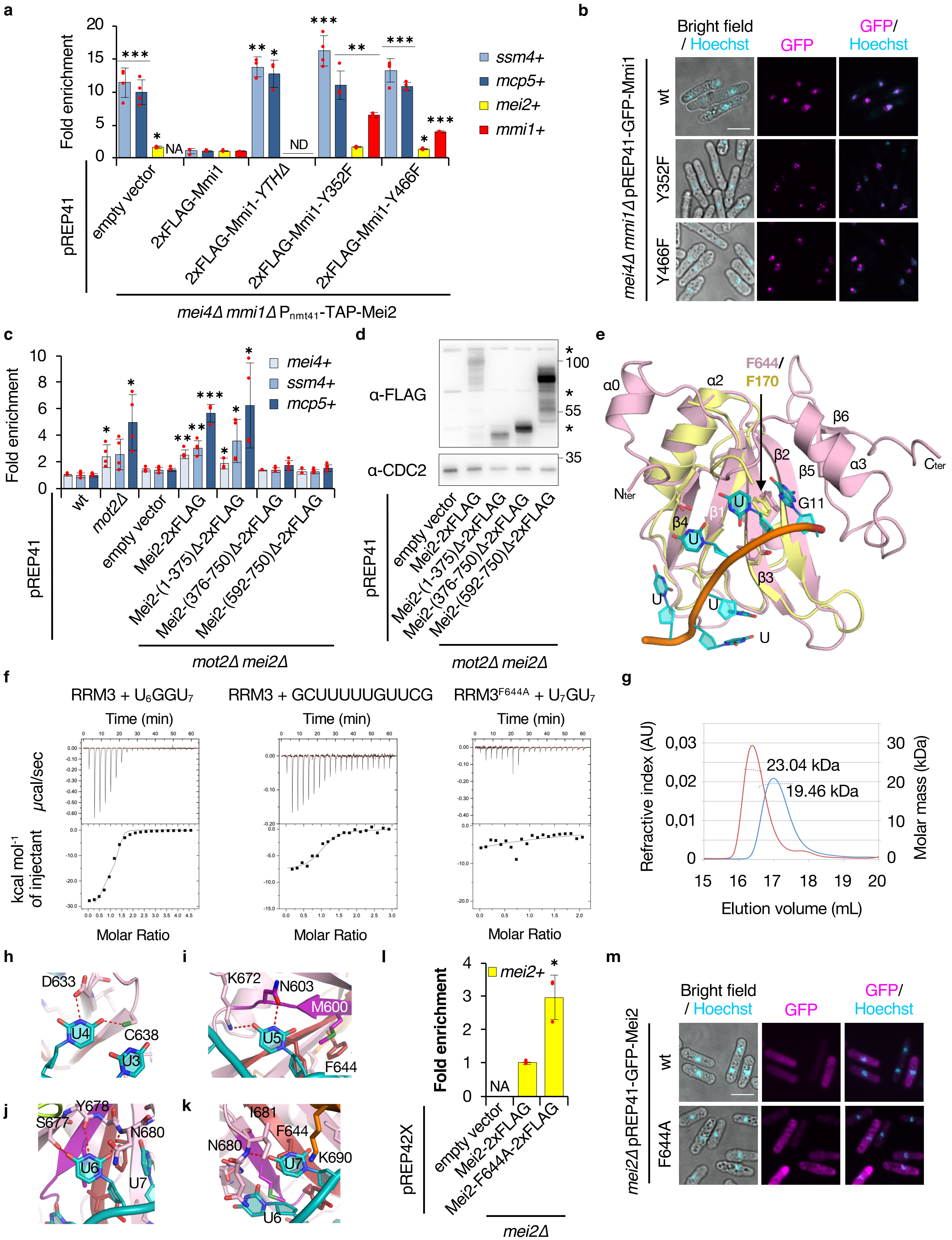

Supplementary Fig. 1 (related to Fig. 1): Mmi1 and Mei2 RNA-binding activities are required for their mutual control . a, RT-qPCR analyses of *ssm4*+, *mcp5*+, *mei2*+ and *mmi1*+ mRNA levels (mean±SD; n=3 or 4; normalized to *act1*+ and relative to 2xFLAG-

Supplementary Fig. 1 (continued): tagged *wt* Mmi1) in cells of the indicated genetic backgrounds. Student's *t*-test (two-tailed) was used to calculate *p*-values. Relative to cells expressing pREP41-2xFLAG-Mmi1, $p_{ssm4+/mcp5+/mei2+/mmi1+}$= 0.00217 / 0.00237 / 0.017 / NA (empty vector); 0.00327 / 0.01007 / ND / ND (pREP41-2xFLAG-Mmi1-YTHΔ); 0.00073 / 0.00256 / 0.00178 / 0.00114 (pREP41-2xFLAG-Mmi1-Y352F); 0.00054 / 0.00004 / 0.01346 / 0.00013 (pREP41-2xFLAG-Mmi1-Y466F) (0.05>*>0.01; 0.01>**>0.001; ***<0.001). NA, not applicable. ND, not determined. Individual data points are represented by red circles. **b**, Representative live cell microscopy images of GFP-tagged *wt* and mutant Mmi1. DNA was stained with Hoechst. The white scale bar represents 10 μm. **c**, RT-qPCR analyses of *mei4*+, *ssm4*+ and *mcp5*+ meiotic mRNA levels (mean±SD; n=4; normalized to *act1*+ and relative to *wt*) in cells of the indicated genetic backgrounds. Student's *t*-test (two-tailed) was used to calculate *p*-values. Relative to *wt* cells, $p_{mei4+/ssm4+/mcp5+}$= 0.04305 / 0.07036 / 0.03144 (*mot2Δ*); 0.00191 / 0.00311 / 0.00056 (pREP41-Mei2-2xFLAG); 0.02121 / 0.04912 / 0.04603 (pREP41-Mei2-(1-375)Δ-2xFLAG) (0.05>*>0.01; 0.01>**>0.001; ***<0.001). Individual data points are represented by red circles. **d**, Western blots showing total levels of 2xFLAG-tagged constructs of Mei2. Anti-CDC2 antibody was used as loading control. Stars denote non-specific bands. **e**, Superimposition of RRM3^{Mei2} (pink) and *Drosophila melanogaster* RRM1^{Sxl} (yellow) bound to a U-rich RNA fragment (RNA phosphate backbone in orange and bases in blue; PDB code: 4QQB). RRM3^{Mei2} helix α0 is part of the N-terminal extension (residues 580 to 593) while helix α3 and β-strands β5 and β6 correspond to the C-terminal extension (residues 684 to 727). Only RRM1^{Sxl} domain and a fragment of the RNA are shown. Side chains from Mei2 F644 and Sxl F170, which belong to the GYA<u>F</u> signature from RRM RNP1 motif that is strictly conserved among Mei2-like orthologues and Sxl, are shown as sticks. **f**, Upper panels: ITC data obtained by injecting various RNAs to RRM3^{Mei2} or RRM3$^{Mei2\text{-}F644A}$. Lower panels: Fitting of the binding curves using a single binding site model. **g**, RRM3^{Mei2} is monomeric in solution and binds a single RNA molecule according to SEC-MALLS. Shown are RRM3^{Mei2} (blue) and RRM3^{Mei2}-RNA (red) elution profiles and the associated molecular weight (dashed lines). The molecular weight of the RNA alone is 4.29 kDa. **h-k**, Detailed representation of U4 (**h**), U5 (**i**), U6 (**j**) and U7 (**k**) binding modes, highlighting the hydrogen bonds (red dashed lines) contributing to the specificity for U. **l**, RT-qPCR analyses of *mei2*+ mRNA levels (mean±SD; n=3; normalized to *act1*+ and relative to 2xFLAG-tagged *wt* Mei2) in cells of the indicated genetic backgrounds. Student's *t*-test (two-tailed) was used to calculate *p*-values. Between cells expressing pREP42X-Mei2-2xFLAG and pREP42X-Mei2-F644A-2xFLAG, *p*=0.03583 (0.05>*>0.01). Individual data points are represented by red circles. NA, not applicable. **m**, Representative live cell microscopy images of GFP-tagged *wt* and mutant Mei2. DNA was stained with Hoechst. The white scale bar represents 10 μm.

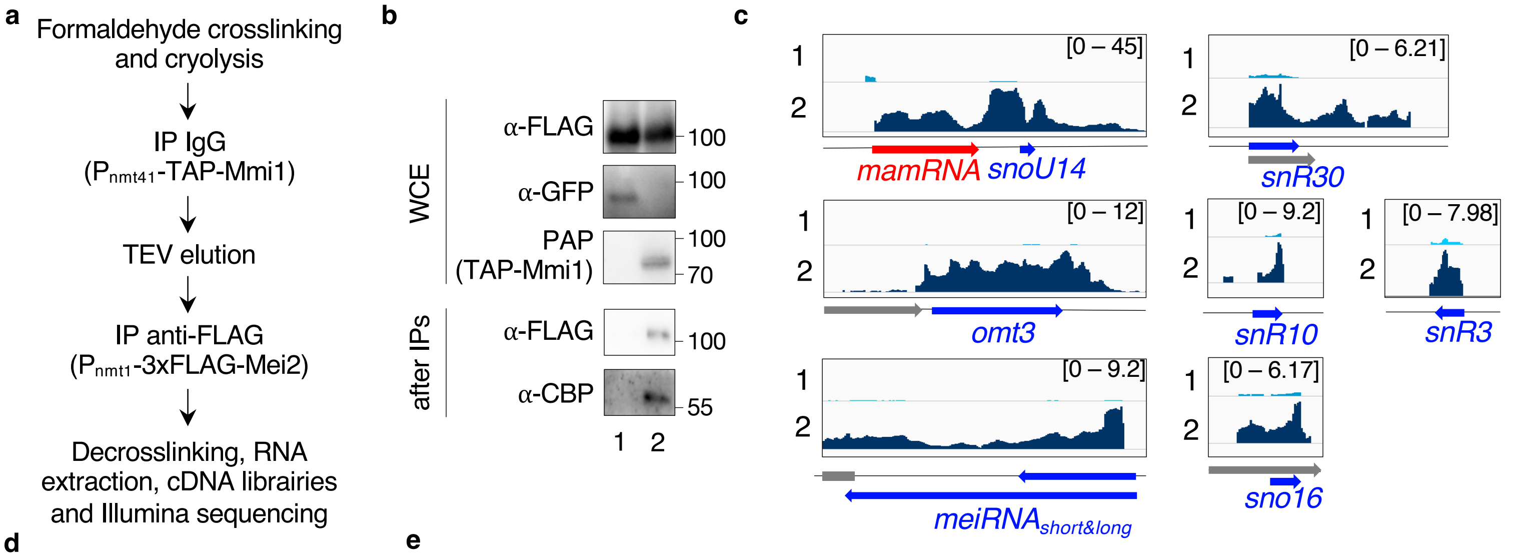

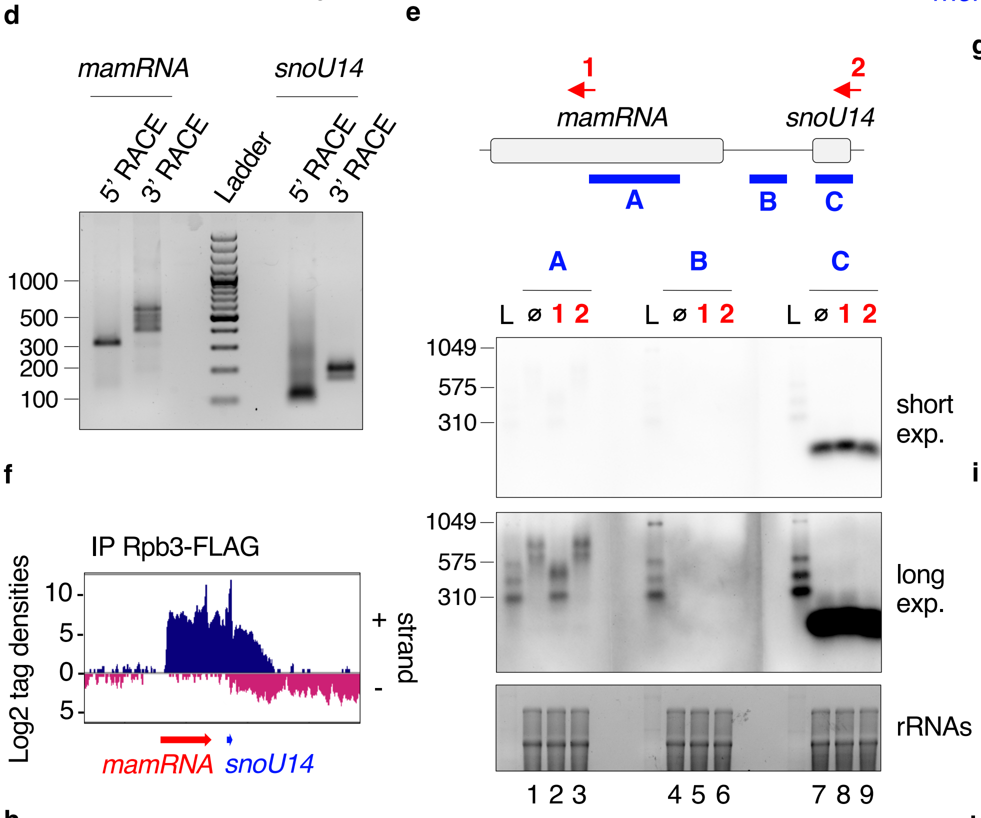

g

Identity	Length (NTs)	CP	Label
snoU14	112	0.018	NC
mamRNA	**678**	**0.022**	**NC**
omt3	1001	0.023	NC
meiRNA	1562	0.039	NC
act1+	1833	1	C

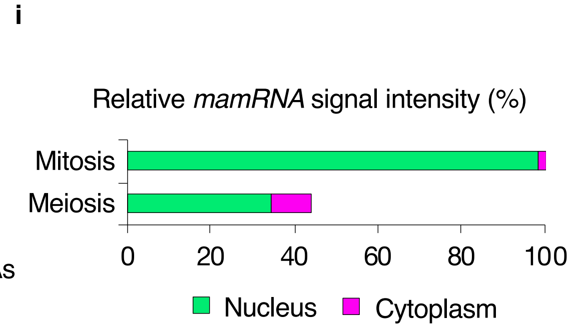

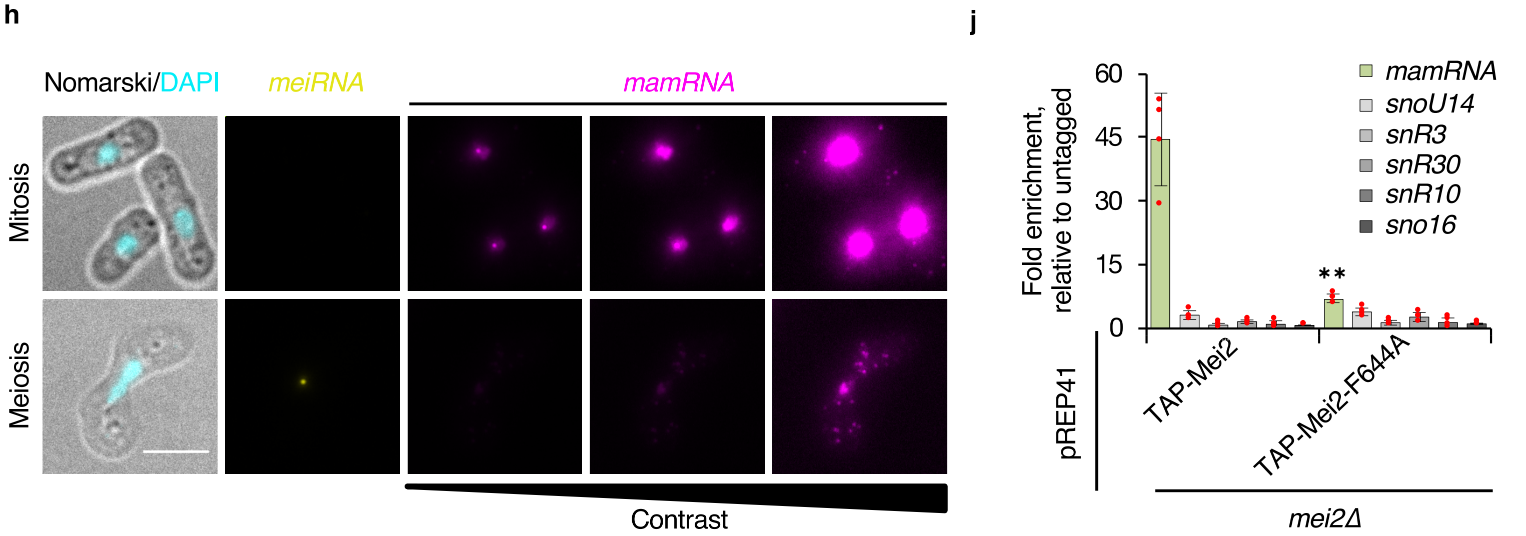

Supplementary Fig. 2 (related to Fig. 2): Identification of *mamRNA*, an Mmi1 and Mei2-scaffolding lncRNA. a, Scheme depicting the main steps of the seqRIP-seq procedure. To capture transient protein-RNA interactions and to increase the yield of RNA recovery following the two immunoprecipitation steps, a mild formaldehyde crosslinking step was performed. **b**, Western blots showing total and immunoprecipitated levels of Mmi1 and Mei2 in strains used for seqRIP-seq (1: P_{nmt41}-GFP-Mmi1 P_{nmt1}-3xFLAG-Mei2, negative control / 2: P_{nmt41}-TAP-Mmi1 P_{nmt1}-3xFLAG-Mei2). PAP, anti-GFP, anti-CBP and anti-FLAG antibodies were used to

Supplementary Fig. 2 (continued): detect tagged proteins in the total (WCE) and immunoprecipitated (IP IgG + IP anti-FLAG) fractions. **c**, seqRIP-seq profiles of 8 ncRNAs simultaneously bound by Mmi1 and Mei2 (red dots in **Fig. 2a**). The numbers in square brackets indicate the range of reads corresponding to the enrichments of immunoprecipitated over total RNAs. 1 and 2 refer to strain genotypes as described in **b**. **d**, 5' and 3' RACE analyses of *mamRNA* and *snoU14*. cDNA samples were loaded on a BET-containing 2% agarose gel. Size values of 100 bp DNA ladder are shown on the left side of the gel. **e**, Northern blot analysis of *mamRNA* and *snoU14*. Upper panel: Scheme depicting the *mamRNA* and *snoU14*-encoding genomic locus. The red arrows represent the oligonucleotides used for RNAseH cleavage and the blue rectangles indicate the Northern probes used for transcript detection. Lower panel: Northern blotting showing RNAseH-dependent cleavage of *mamRNA* (probe A) with oligonucleotide 1 but not 2 (lanes 2 and 3), and of *snoU14* (probe C) with oligonucleotide 2 but not 1 (lanes 8 and 9). Samples from reactions performed without oligonucleotides were loaded as controls (lanes 1, 4 and 7). Note that the probe B, designed between *mamRNA* and *snoU14*, does not detect RNA species. **f**, NET-seq profile of the *mamRNA* and *snoU14*-encoding genomic locus. Shown are log2 tag densities corresponding to RNA PolII-immunoprecipitated (FLAG-tagged Rpb3 subunit) RNAs. Tags from the + strand are colored in blue and those from the – strand in magenta. The positions of *mamRNA* and *snoU14* are depicted below the profiles. **g**, Analysis of *mamRNA* coding potential with CPC2 software. Table showing the identity, length (in nucleotides), coding probability (CP) and label (NC=noncoding; C=coding) of 4 ncRNAs (*snoU14*, *mamRNA*, *meiRNA*, *omt3*) and 1 mRNA (*act1*+). **h**, Representative images of *meiRNA* and *mamRNA* detected by smFISH in *wt* mitotic and meiotic cells (horsetail stage of meiotic prophase I). DNA was stained with DAPI. Images are shown as maximum-intensity projections of Z-stacks. Distinct contrast adjustments are shown to compare *mamRNA* signals. The white scale bar represents 5 μm. **i**, Quantification of smFISH analyses shown in panel **h**. Distribution of *mamRNA* signal intensities (integrated densities) from areas corresponding to the nucleus and cytoplasm of mitotic (n=53) and meiotic (n=52) cells, expressed as percentages of the total signal in mitosis. **j**, Fold enrichments (mean±SD; n=4; relative to untagged) of 5 sn/snoRNAs and *mamRNA* upon pulldown of TAP-tagged *wt* or mutant (F644A) Mei2. Student's t-test (two-tailed) was used to calculate *p*-values. Between cells expressing pREP41-TAP-Mei2 and pREP41-TAP-Mei2-F644A, p=0.00608 (mamRNA) (0.01>**>0.001). Individual data points are represented by red circles.

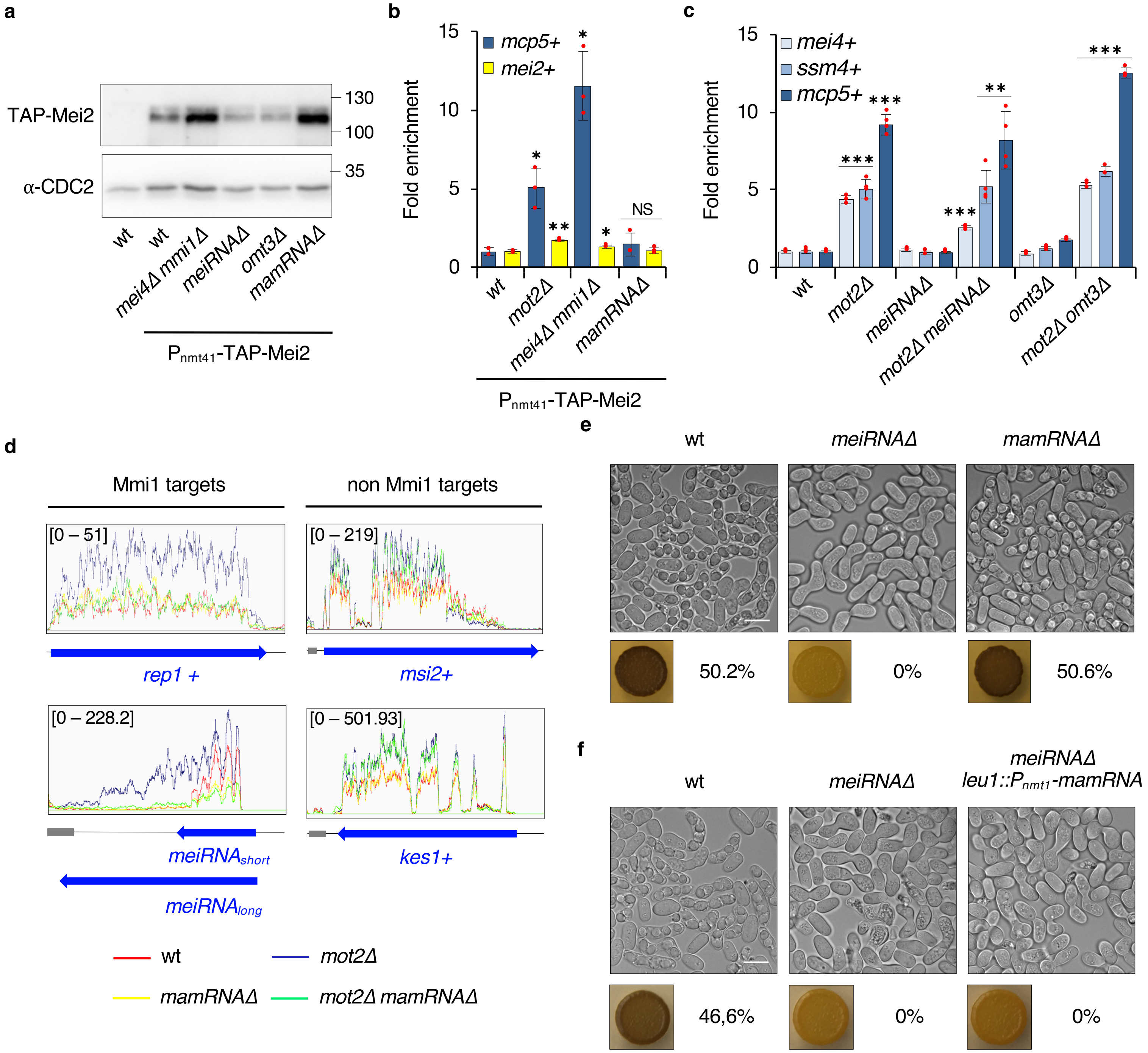

Supplementary Fig. 3 (related to Fig. 3): *mamRNA* mediates the Mmi1-Mei2 mutual control. a, Western blots showing total TAP-tagged Mei2 levels in cells of the indicated genetic backgrounds. Anti-CDC2 antibody was used as loading control. **b** and **c**, RT-qPCR analyses of *mei2*+, *mei4*+, *ssm4*+ and *mcp5*+ meiotic mRNA levels (mean±SD; n=3 or 4; normalized to *act1*+ and relative to *wt*) in cells of the indicated genetic backgrounds. Student's *t*-test (two-tailed) was used to calculate *p*-values. Relative to *wt* cells in **b**, $p_{mcp5+/mei2+}$= 0.02856 / 0.0014 (*mot2Δ*); 0.01327 / 0.03395 (*mei4Δ mmi1Δ*); 0.40797 / 0.74937 (*mamRNAΔ*). Relative to *wt* cells in **c**, $p_{mei4+/ssm4+/mcp5+}$= 0.00004 / 0.0008 / 0.00013 (*mot2Δ*); 7.4 x 10^{-6} / 0.00386 / 0.00443 (*mot2Δ meiRNAΔ*); 0.00012 / 0.00052 / 0.00018 (*mot2Δ omt3Δ*). (0.05>*>0.01; 0.01>**>0.001; ***<0.001). NS, not significant. Individual data points are represented by red circles. **d**, RNA-seq profiles of 4 genes upregulated in *mot2Δ* cells in a *mamRNA*-dependent (left; Mmi1 targets) or -independent (right; non Mmi1 targets) manner (mean; n=2). **e** and **f**, Mating/sporulation efficiencies of the indicated homothallic strains (% tetrads), as determined by iodine staining and live cell imaging. In **e**, n_{wt}=496 cells; $n_{meiRNAΔ}$=500; $n_{mamRNAΔ}$=502. In **f**, n_{wt}=785 cells; $n_{meiRNAΔ}$=762; $n_{meiRNAΔ\ leu1::Pnmt1\text{-}mamRNA}$=730. White scale bars represent 10 μm.

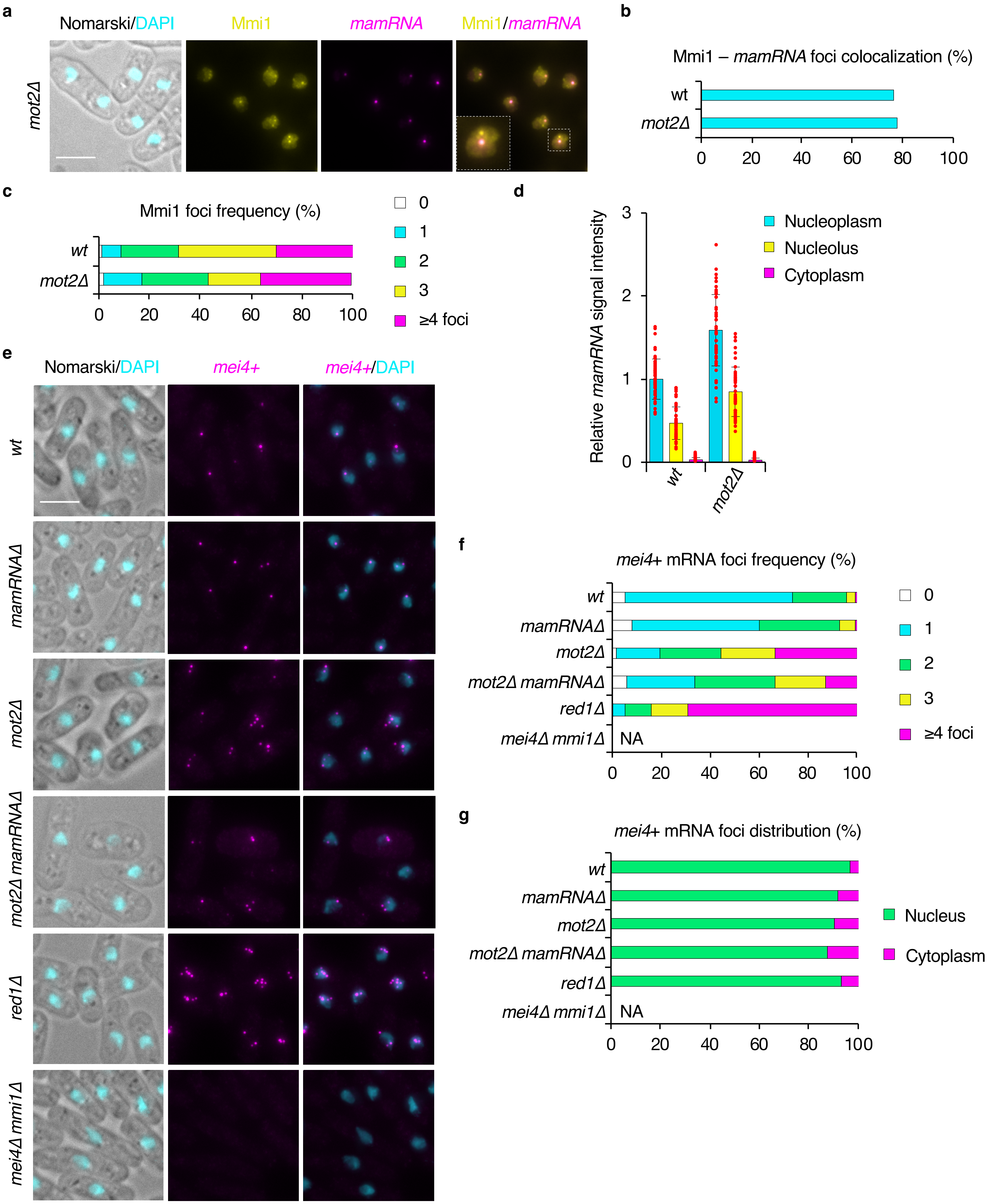

Supplementary Fig. 4 (related to Fig. 4): Subcellular localization of Mmi1, *mamRNA* and meiotic mRNAs. a, Representative images of *mamRNA* detected by smFISH in *mot2Δ* cells. DNA was stained with DAPI. GFP-tagged Mmi1 was visualized in parallel. Images are shown as maximum-intensity projections of Z-stacks. The white scale bar represents 5 *μ*m. **b**, Quantification of Mmi1 and *mamRNA* colocalization in *wt* and *mot2Δ* cells, expressed as the fraction of *mamRNA* spots colocalizing with Mmi1 nuclear bodies (%; n_{wt}=703 nuclei; $n_{mot2\Delta}$= 579). **c**, Quantification of Mmi1 nuclear bodies. Shown is the distribution of Mmi1 foci frequency per nucleus in *wt* and *mot2Δ* cells (%; n_{wt}=549 nuclei; $n_{mot2\Delta}$=592). **d**, Quantification of smFISH analyses. Shown are the *mamRNA*

Supplementary Fig. 4 (continued): signal intensities from areas corresponding to the nucleoplasm, nucleolus and cytoplasm in *mot2Δ* cells (integrated densities relative to nucleoplasmic average; mean±SD; n=50 cells). Data for *wt* cells are also displayed in **Fig. 2d**. Individual data points are represented by red circles. **e**, Representative images of *mei4+* mRNAs detected by smFISH in cells of the indicated genetic backgrounds. DNA was stained with DAPI. Images are shown as maximum-intensity projections of Z-stacks. The white scale bar represents 5 μm. **f** and **g**, Quantifications of smFISH analyses shown in **e**. **f**, Distribution of *mei4+* mRNA foci frequency per cell (n_{wt}= 711 cells; $n_{mamRNA\Delta}$=703; $n_{mot2\Delta}$=502; $n_{mot2\Delta mamRNA\Delta}$=579; $n_{red1\Delta}$=479). **g**, Distribution of *mei4+* mRNA foci localization (n_{wt}=895 foci; $n_{mamRNA\Delta}$=972; $n_{mot2\Delta}$=1531; $n_{mot2\Delta mamRNA\Delta}$=1228; $n_{red1\Delta}$=1016). NA, not applicable.

Supplementary Table 1: Structural characterization of RRM3^{Mei2}

**Data collection**			
	**RRM3^{Mei2} apo**		**RRM3^{Mei2}-RNA**
	Se-SAD	High resolution	
Space group	$P4_1$	$P4_1$	I222
Unit cell parameters	75.1Å; 75.1Å; 67.8Å; 90°; 90°; 90°	75.5Å; 75.5Å; 70.8Å; 90°; 90°; 90°	75.3Å; 81.5Å; 81.4Å; 90°; 90°; 90°
Wavelength (Å)	0.9080105	0.9080105	0.9080105
Resolution (Å)	41.8-2.5 (2.59-2.5)	42.63-1.9 (1.94-1.9)	40.5-2.65 (2.78-2.65)
R_{merge} (%)	8.1 (120.4)	5.8 (84.8)	25.4 (167.3)
$I / \sigma I$	14 (1.3)	11.1 (1.3)	6.4 (1.2)
Completeness (%)	99.0 (92.4)	99.0 (89.6)	99.6 (97.7)
$CC_{1/2}$ (%)	99.7 (43.1)		98.6 (72.8)
Redundancy	6.5	3.7	11.4
Observed reflections	86530	117825	83891
Unique reflections	13280	31476	7387
**Refinement**			
Resolution (Å)		42.63-1.9	40.7-2.63
R / R_{free} (%)		17.1 / 19.4	22.3 / 29.8
Number of atoms			
Protein / RNA		2380	1199 / 246
Water		187	30
Others		92	
B-factors (Å^2)			
Protein / RNA		44.3	61.8 / 66.7
Water		46.2	52.2
Others		76.3	
R.m.s deviations			
Bond lengths (Å)		0.01	0.01
Bond angles (°)		0.98	1.07
PDB code		6YYL	6YYM

Supplementary Table 2: Thermodynamic parameters for RNA binding to RRM3^{Mei2}

Protein	RNA	Kd (nM)	N	ΔH (kcal/mol)	TΔS (kcal/mol)
RRM3^{Mei2} (9 μM)	U_7GU_7 (140 μM)	145 ± 26	1	-21.31 ± 0.33	-12.13
RRM3^{Mei2} (9 μM)	U_6GGU_7 (220 μM)	435 ± 111.8	1	-29.07 ± 0.9	-20.54
RRM3^{Mei2} (9 μM)	U_{15} (130 μM)	1432.6 ± 194.2	1	-15.82 ± 0.43	-7.97
RRM3^{Mei2} (9 μM)	GCUUUUUGUUCG (140 μM)	555 ± 166.5	1	-7.7 ± 0.3	0.69
RRM3$^{Mei2\text{-}F644A}$ (9 μM)	U_7GU_7 (140 μM)	ND	ND	ND	ND

ND: not detectable

Supplementary Table 3: Hydrogen bonds at the RRM3^{Mei2}-RNA interface

RRM3^{Mei2}		RNA	
**Residue**	**Atom**	**Base**	**Atom**
K607	N	**C2**	O2
F668	O	**C2**	O2’
N606	N	**U3**	OP2
S670	Og	**U3**	OP1
C638	Sg	**U4**	O4
K672	Nz	**U4**	OP2
D633	Od1	**U4**	N3
K672	Nz	**U5**	O4
N603	Od1	**U5**	N3
Y678	O	**U6**	N3
S677	Og	**U6**	O4
N680	N	**U6**	O2
N680	Nd2	**U6**	O2’
I681	N	**U7**	O4
R631	Nh1	**G8**	O4’
I632	O	**G8**	N2
Y629	Oh	**G8**	N2
I632	O	**G8**	N1
F634	N	**G8**	O6
C695	Sg	**G8**	O2’
Y629	Oh	**G8**	OP2
D633	Od1	**G12**	N2

Supplementary Table 4: ***S. pombe*** **strains used in this study**

Strain	Genotype	Source
PR040	h90, *ura4-DS/E, ade6-M210, leu1-32, mat3M::ura4+*	Simonetti et al., 2017
PR162	h90, *ura4-D18, ade6-M210, leu1-32, mat3M(EcoRV)::ade6+*	JP Javerzat
PR411	PR040, mei4*::nat*^{R}*MX* mmi1*::hph*^{R}*MX*	Simonetti et al., 2017
PR525	PR162, mot2*::nat*^{R}*MX*	This study
PR597	PR040, *kan*^{R}*MX*::P$_{nmt41}$-3xFLAG-Mmi1	Simonetti et al., 2017
PR617	PR040, sme2*::hph*^{R}*MX*	This study
PR640	PR040, mot2*::nat*^{R}*MX* sme2*::hph*^{R}*MX*	This study
PR675	PR040, *kan*^{R}*MX*::P$_{nmt41}$-TAP-Mei2	Simonetti et al., 2017
PR676	PR040, Mei2-GFP::*kan*^{R}*MX*	Simonetti et al., 2017
PR718	PR040, sme2*::hph*^{R}*MX kan*^{R}*MX*::P$_{nmt41}$-TAP-Mei2	This study
PR720	PR040, mot2*::nat*^{R}*MX kan*^{R}*MX*::P$_{nmt41}$-TAP-Mei2	Simonetti et al., 2017
PR726	PR040, mei4*::nat*^{R}*MX* mmi1*::hph*^{R}*MX kan*^{R}*MX*::P$_{nmt41}$-TAP-Mei2	Simonetti et al., 2017
PR792	PR040, mot2*::nat*^{R}*MX*	Simonetti et al., 2017
PR793	PR040, mot2*::nat*^{R}*MX kan*^{R}*MX*::P$_{nmt41}$-3xFLAG-Mmi1	This study
PR808	PR040, pREP41*::LEU2*	This study
PR810	PR040, mot2*::nat*^{R}*MX* pREP41*::LEU2*	This study
PR911	PR040, mot2*::nat*^{R}*MX* mei2*::hph*^{R}*MX* pREP41*::LEU2*	This study
PR965	PR040, mot2*::nat*^{R}*MX* mei2*::hph*^{R}*MX* pREP41-Mei2-(376-750)Δ-2xFLAG*::LEU2*	This study
PR966	PR040, mot2*::nat*^{R}*MX* mei2*::hph*^{R}*MX* pREP41-Mei2-(592-750)Δ-2xFLAG*::LEU2*	This study
PR968	PR040, mot2*::kan*^{R}*MX*	Simonetti et al., 2017
PR980	PR040, mot2*::nat*^{R}*MX* mei2*::hph*^{R}*MX* pREP41-Mei2-2xFLAG*::LEU2*	This study
PR981	PR040, mot2*::nat*^{R}*MX* mei2*::hph*^{R}*MX* pREP41-Mei2-(1-375)Δ-2xFLAG*::LEU2*	This study

PR1021	PR040, omt3::*hph*^{R}*MX*	This study
PR1026	PR040, mei4::*nat*^{R}*MX* mmi1::*hph*^{R}*MX* *kan*^{R}*MX*::P_{nmt41}-TAP-Mei2 pREP41::*LEU2*	This study
PR1027	PR040, mot2::*nat*^{R}*MX* omt3::*hph*^{R}*MX*	This study
PR1033	PR040, *kan*^{R}*MX*::P_{nmt41}-GFP-Mmi1	This study
PR1034	PR040, mei2::*hph*^{R}*MX* pREP41::*LEU2*	This study
PR1035	PR040, mei4::*nat*^{R}*MX* mmi1::*hph*^{R}*MX* *kan*^{R}*MX*::P_{nmt41}-TAP-Mei2 pREP41-2xFLAG-Mmi1::*LEU2*	This study
PR1036	PR040, mot2::*nat*^{R}*MX* *kan*^{R}*MX* ::P_{nmt41}-GFP-Mmi1	This study
PR1037	PR040, mei4::*nat*^{R}*MX* mmi1::*hph*^{R}*MX* *kan*^{R}*MX*::P_{nmt41}-TAP-Mei2 pREP41-2xFLAG-Mmi1-YTHΔ::*LEU2*	This study
PR1038	PR040, mei4::*nat*^{R}*MX* mmi1::*hph*^{R}*MX* *kan*^{R}*MX*::P_{nmt41}-TAP-Mei2 pREP41-2xFLAG-Mmi1-Y352F::*LEU2*	This study
PR1039	PR040, mei4::*nat*^{R}*MX* mmi1::*hph*^{R}*MX* *kan*^{R}*MX*::P_{nmt41}-TAP-Mei2 pREP41-2xFLAG-Mmi1-Y466F::*LEU2*	This study
PR1056	PR040, mei4::*nat*^{R}*MX* mmi1::*hph*^{R}*MX* pREP41-2xFLAG-Mmi1::*LEU2*	This study
PR1105	PR040, omt3::*hph*^{R}*MX* *kan*^{R}*MX*::P_{nmt41}-TAP-Mei2	This study
PR1122	PR040, *nat*^{R}*MX*::P_{nmt1}-3xFLAG-Mei2 *kan*^{R}*MX*::P_{nmt41}-TAP-Mmi1	This study
PR1141	PR162, pREP42X::*URA4*	This study
PR1142	PR162, mei2::*hph*^{R}*MX* pREP42X::*URA4*	This study
PR1143	PR162, mei2::*hph*^{R}*MX* pREP42X-Mei2-2xFLAG::*URA4*	This study
PR1144	PR162, mei2::*hph*^{R}*MX* pREP42X-Mei2-F644A-2xFLAG::*URA4*	This study
PR1147	PR162, mot2::*nat*^{R}*MX* mei2::*kan*^{R}*MX* pREP42X::*URA4*	This study
PR1149	PR162, mot2::*nat*^{R}*MX* mei2::*kan*^{R}*MX* pREP42X-Mei2-F644A-2xFLAG::*URA4*	This study
PR1159	PR162, mot2::*nat*^{R}*MX* pREP42X::*URA4*	This study
PR1163	PR162, mot2::*nat*^{R}*MX* mei2::*kan*^{R}*MX* pREP42X-Mei2-2xFLAG::*URA4*	This study
PR1168	PR040, *nat*^{R}*MX*::P_{nmt1}-3xFLAG-Mei2 *kan*^{R}*MX*::P_{nmt41}-GFP-Mmi1	This study
PR1208	PR040, *hph*^{R}*MX*::P_{nmt41}-GFP-Mmi1	This study
PR1225	PR040, mot2::*nat*^{R}*MX* *hph*^{R}*MX*::P_{nmt41}-GFP-Mmi1	This study

PR1228	PR040, mamRNA::*nat*^{R}*MX*	This study
PR1238	PR040, mamRNA::*nat*^{R}*MX kan*^{R}*MX*::P$_{nmt41}$-TAP-Mei2	This study
PR1239	PR040, mot2*::kan*^{R}*MX* mamRNA::*nat*^{R}*MX*	This study
PR1242	PR040, mamRNA::*nat*^{R}*MX hph*^{R}*MX*::P$_{nmt41}$-GFP-Mmi1	This study
PR1310	PR040, mot2*::kan*^{R}*MX* mamRNA::*nat*^{R}*MX hph*^{R}*MX*::P$_{nmt41}$-GFP-Mmi1	This study
PR1316	PR040, mei4*::nat*^{R}*MX* mmi1*::hph*^{R}*MX* pREP41*::LEU2*	This study
PR1317	PR040, mei4*::nat*^{R}*MX* mmi1*::hph*^{R}*MX* pREP41-2xFLAG-Mmi1-Y352F*::LEU2*	This study
PR1318	PR040, mei4*::nat*^{R}*MX* mmi1*::hph*^{R}*MX* pREP41-2xFLAG-Mmi1-Y466F*::LEU2*	This study
PR1338	PR040, mot2::*nat*^{R}*MX* mamRNA::*hph*^{R}*MX kan*^{R}*MX*::P$_{nmt41}$-TAP-Mei2	This study
PR1340	PR040, mei4*::nat*^{R}*MX* mmi1*::hph*^{R}*MX* pREP41-GFP-Mmi1*::LEU2*	This study
PR1341	PR040, mei4*::nat*^{R}*MX* mmi1*::hph*^{R}*MX* pREP41-GFP-Mmi1-Y352F*::LEU2*	This study
PR1342	PR040, mei4*::nat*^{R}*MX* mmi1*::hph*^{R}*MX* pREP41-GFP-Mmi1-Y466F*::LEU2*	This study
PR1349	PR040, mamRNA::*hph*^{R}*MX kan*^{R}*MX*::P$_{nmt41}$-TAP-Mei2	This study
PR1377	PR040, sme2*::hph*^{R}*MX* pREP41-GFP-Mei2*::LEU2*	This study
PR1393	PR040, sme2*::hph*^{R}*MX* pREP41-GFP-Mei2-NLS*::LEU2*	This study
PR1394	PR040, sme2*::hph*^{R}*MX* mamRNA::*nat*^{R}*MX* pREP41-GFP-Mei2-NLS*::LEU2*	This study
PR1407	PR040, mei2*::hph*^{R}*MX* pREP41-TAP-Mei2*::LEU2*	This study
PR1408	PR040, mei2*::hph*^{R}*MX* pREP41-TAP-Mei2-F644A*::LEU2*	This study
PR1436	PR040, red1::*nat*^{R}*MX kan*^{R}*MX*::P$_{nmt41}$-GFP-Mmi1	This study
PR1455	PR040, mamRNA::*hph*^{R}*TK kan*^{R}*MX*::P$_{nmt41}$-TAP-Mei2	This study
PR1459	PR040, mot2*::kan*^{R}*MX* mamRNA::*hph*^{R}*TK*	This study
PR1525	h90, *ura4-DS/E, ade6-M210, mat3M::ura4+,* mamRNA::*nat*^{R}*MX kan*^{R}*MX*::P$_{nmt41}$-TAP-Mei2 *leu1*::P$_{nmt1}$-snoU14	This study
PR1526	h90, *ura4-DS/E, ade6-M210, mat3M::ura4+,* mamRNA::*nat*^{R}*MX kan*^{R}*MX*::P$_{nmt41}$-TAP-Mei2 *leu1*:: P$_{nmt1}$-mamRNA	This study
PR1611	h90, *ura4-DS/E, ade6-M210, mat3M::ura4+,* sme2::*hph*^{R}*MX leu1*::P$_{nmt1}$-mamRNA	This study

Supplementary Table 5: oligonucleotides used in this study

Primers	Sequence	Assays	Figures
P249: *mei4+* fwd	5’-TGGATCAGATCCGTGGAATC-3’	qPCR	1j, 3c, 4e, S1c, S3c
P250: *mei4+* rev	5’-AACGCTCGATTAGAAGGCAT-3’		
P253: *act1+* fwd	5’-AACCCTCAGCTTTGGGTCTT-3’		1c, 1h, 1j, 3c, 4e, S1a, S1c, S1l, S3b, S3c
P254: *act1+* rev	5’-TTTGCATACGATCGGCAATA-3’		
P325: *ssm4+* fwd	5’-ACACAGTTTACGGGATTCTA-3’		1c, 1j, 3c, S1a, S1c, S3c
P326: *ssm4+* rev	5’-GATTGTGATGAAAACTGGGT-3’		
P337: *mmi1*+ rev	5’-AAGGCCATTCGTAGGAGTGA-3’		S1a
P451: *mmi1*+ fwd	5’-CACCTCTGAAAAGACCTGCA-3’		
P573: *meiRNA* fwd	5’-GGATGAATAGTAGCTTAGAT-3’		1h, 2f, 4e
P574: *meiRNA* rev	5’-GCTTTCAAGGATAACAATGC-3’		
P607: *mcp5+* fwd	5’-AGACGTATTCACCTTACCTC-3’		1c, 1j, 3c, 4e, S1a, S1c, S3b, S3c
P608: *mcp5+* rev	5’-GTTTCCCATCATGACATGTT-3’		
P881: *mei2*+ rev	5’-GGGATTCTGAGAGAACAGAA-3’		S1a, S1l, S3b
P1010: *mei2*+ fwd	5’-GAGTTGGTGAACGGAAAGTA-3’		
P1194: *omt3* rev	5’-GTCACGTACATCAAACATCAT-3’		2f, 2g
P1207: *omt3* fwd	5’-AGGTCTTATAAGAAACATATGTGT-3’		
P1208: *mamRNA* fwd	5’-TGGGATTAAGTCATCTTGGTG-3’		2f, 2g, S2j
P1209: *mamRNA* rev	5’-GTCAAAAAGATACCACAGCA-3’		
P1284: *snoU14* rev	5’-CATCCAAAGGAAGGACTATG-3’		S2j
P1392: *snoU14* fwd	5’-CAGGTGATGAAATTTCCATTG-3’		
P1470: *sno16* fwd	5’-AGGAGTAAATTAAACTGCCTC-3’		
P1471: *sno16* rev	5’-CAAGGAAATTACAGCTGCTT-3’		
P1472: *snR3* fwd	5’-GCATTCGTATTTGCTATTCAC-3’		
P1473: *snR3* rev	5’-TATGTGGACCTTCTGACACT-3’		
P1474: *snR10* fwd	5’-GAAAACCATCTCACCCAATC-3’		
P1475: *snR10* rev	5’-GGATTCAAAACTTGATGCAGT-3’		
P1476: *snR30* fwd	5’-GTGTAGAGGTTGACAAGAGT-3’		
P1477: *snR30* rev	5’-TACGCAGCATCCTTTAGTC-3’		

P1324: *mamRNA* rev	5’-CCATACACGACAGACATACTGTACAATC-3’	RNAseH cleavage	S2e
P1450: *snoU14* rev	5’-AACATCAGACATCCAAAGGAAGG-3’		
P1420: *mamRNA* fwd	5’-CTGGGATTAAGTCATCTTGGTGCAG-3’	Northern probes (T7 promoter)	2b, S2e
P1421: *mamRNA* rev	5’-TAATACGACTCACTATAGGGGAACAGCTTCGTTGTACTCAGACATC-3’		
P234: *adh1+* fwd	5’-AACGTCAAGTTCGAGGAAGTCC-3’		2b
P1460: *adh1+* rev	5’-TAATACGACTCACTATAGGGGTCTCCTCAGCCTTCATACAGTACTC-3’		
P1424: *U3B* fwd	5’-GTCCGGCTTGGGTTTCCTAAC-3’		
P1425: *U3B* rev	5’-TAATACGACTCACTATAGGGCGTCAGAAAACACCAGCTGC-3’		
P1448: *snoU14* fwd	5’-ATCAACAGGTGATGAAATTTCCATTG-3’		S2e
P1450: *snoU14* rev	5’-TAATACGACTCACTATAGGGAACATCAGACATCCAAAGGAAGG-3’		
P1511: between *mamRNA* and *snoU14* fwd	5’-CTTGCTATGCTTAAACTCGC-3’		
P1512: between *mamRNA* and *snoU14* rev	5’-TAATACGACTCACTATAGGGAATCTCCACCATAACTCGTAC-3’		
P1323: *mamRNA* rev	5’-TACAAGGGATCCCTGCACCAAGATGACTTAATCCCAG-3’	5’ RACE (extension, BamHI site)	S2d
P1324: *mamRNA* rev-nested	5’-TACAAGGGATCCCCATACACGACAGACATACTGTACAATC-3’		
P1492: *snoU14* rev	5’-TACAAGGGATCCAACATCAGACATCCAAAGGAAGG-3’		
P1493: *snoU14* rev-nested	5’-TACAAGGGATCCAATTGGGTTCGTATGCCGC-3’		
P1325: *mamRNA* fwd	5’-TACAAGGGATCCGGATGGTTCTCCTTCAGGCTCTTGG-3’	3’ RACE (extension, BamHI site)	
P1491: *snoU14* fwd	5’-TACAAGGGATCCATCAACAGGTGATGAAATTTCCATTG-3’		
oMG432: Mei2-[579-750] WT	5’-CCTGCCCGGGTTCAGATAGAAATTCTGTCGATTATGC-3’	Cloning for expression in *E. coli*	1e, 1f, S1e, S1f, S1g, Tables S1 and S2
oMG429: Mei2-[579-750] WT	5’-GCCGGCGGCCGCTCAACATTTGCTTGCAGTTGG-3’		
oMG486: Mei2-[579-750] F644A	5’-GTAGGATATGCGGCTATAAACTTTATTGAACCTCAATCTA-3’		
oMG487: Mei2-[579-750] F644A	5’-AAGTTTATAGCCGCATATCCTACATTGCATTTATTAACAA-3’		

Supplementary Table 6: smFISH probes used in this study

Target RNA	Probe	Sequence
meiRNA	#1	5’-ATACCCACTAAGTCTGTTTA-3’
	#2	5’-CGGCAGAAGATTGACCAACA-3’
	#3	5’-GCATATTCCGTCTTACAATA-3’
	#4	5’-ACCAACTAAAGCGATCTTGC-3’
	#5	5’-GACCATTTCAAAATGTTGCA-3’
	#6	5’-TACCGAATCCAGCTTTTTGA-3’
	#7	5’-CAGAGCTTAGAAGACAAGGT-3’
	#8	5’-TAACTGGACCCCATCAAGAA-3’
	#9	5’-TAAACCAACTTGGGGGTTGG-3’
	#10	5’-TCTAAGCTACTATTCATCCA-3’
	#11	5’-AGTAGATTCCATCAGTCATA-3’
	#12	5’-TGCAGCCAAAAAGTGTACCA-3’
	#13	5’-CATTGTAAGTGCTTTCAAGG-3’
	#14	5’-TTCAGTCATTCGCAAAGTTT-3’
	#15	5’-AGTCGTTTTATTTCTTTTCT-3’
	#16	5’-GTTTCAACAATAGTTCAGGT-3’
	#17	5’-TCTGTTTCAGGAATACGTTT-3’
	#18	5’-TGTTTCGCATCAAACTTTCA-3’
	#19	5’-GCGTTTAAACAAACTGCGGG-3’
	#20	5’-TGGTTTCAGCACGTTTTCAA-3’
	#21	5’-TTGGTTTGCAGGGTTTAACG-3’
	#22	5’-CTTGCTGTGGTTATTGTTTA-3’
mamRNA	#1	5’-CCTTCAGACTCATGGAAGTA-3’
	#2	5’-TGGCGTCCTAGCATATTAAT-3’
	#3	5’-TAAACCTCTTGTCTCCACTA-3’
	#4	5’-GCCTTTCAACATCAGATAAT-3’
	#5	5’-GCCATTTACGATAAGAACCT-3’
	#6	5’-AAGAGCCTGAAGGAGAACCA-3’
	#7	5’-ACACAGATACAGGCGAACGC-3’
	#8	5’-ATCAGACTAGAATCTCTCCA-3’
	#9	5’-GCAACCATACACGACAGACA-3’
	#10	5’-ACCAAGATGACTTAATCCCA-3’
	#11	5’-CCAACACAGTACCAATTACT-3’
	#12	5’-AGGAATATGGTAGGTTTTTC-3’
	#13	5’-AAAGATACCACAGCAAGCGC-3’
	#14	5’-ACTAAGCTTTAACACCCAGT-3’
	#15	5’-AGTATGCGTACGGGATTTAC-3’
	#16	5’-TTCAGTTTGAGTTCAAGGTT-3’
	#17	5’-CGGCGAACATACCTTTACAA-3’
	#18	5’-TACTCAGACATCAATCAGCA-3’
	#19	5’-TATCATCAGAACAGCTTCGT-3’
	#20	5’-CATAGTCGATAGGACGGGTA-3’
	#21	5’-TCCACCTATAGTGGAGACAA-3’
	#22	5’-AGACATCGAAGATACTGGCA-3’

mcp5+ mRNA	#1	5’-CACCTCGTTATCTTGTTTTT-3’
	#2	5’-CTAACGCTTTCGCATAAGCA-3’
	#3	5’-CTTTTGAACCACTTGGTCTT-3’
	#4	5’-ATTTTTTTCCAAGTCGCGTA-3’
	#5	5’-AAGCATCTCCGTAATTCTCA-3’
	#6	5’-ATTTGTGTTCTTCCTTCTTT-3’
	#7	5’-TGTTGCAGAAGGTTTAAGCC-3’
	#8	5’-AATGGGCATTATCCACTGAC-3’
	#9	5’-CCTTAGTCTTCTCTTTCATA-3’
	#10	5’-AGTTTGTTCTTCTCATCGTT-3’
	#11	5’-GCTCGTGAAGGCAAATTCTT-3’
	#12	5’-CATCTATAGTTTCGCCTTTT-3’
	#13	5’-TTTTCATCCTCAGTTACCAA-3’
	#14	5’-TTATCCGAGGAAGACTCCAA-3’
	#15	5’-CTTGCTCCGCATCAAAAGAT-3’
	#16	5’-TGATGGATTTGAGAACGGCC-3’
	#17	5’-CACTCACTAACTCCTGTTTT-3’
	#18	5’-TTTTAGTATCGTCAGCTACC-3’
	#19	5’-TGTCAGCTTTTGTGTTTTCA-3’
	#20	5’-GAGTTTTCTGGTGATACTGC-3’
	#21	5’-GGTTTCATCTTCGATGTCTA-3’
	#22	5’-AGCTGAAAAAGGCCCAAGTT-3’
	#23	5’-TGAGCTTTTAGTCGGTTTGA-3’
	#24	5’-TGGCGAGGTTCAAATTGAGC-3’
	#25	5’-CAGTACTTGCAGACATGTCG-3’
	#26	5’-ACGTCCACCTTTGTTTAATG-3’
	#27	5’-TGGAGATTGTATTTGCACCA-3’
	#28	5’-ATTCTTTTGCAGATTCCACT-3’
	#29	5’-ACATTTGAAAGCGCATCGGT-3’
	#30	5’-TTTTCTTTTCAACCTCGTGG-3’
	#31	5’-GGATTACTATAGTGGCGCAA-3’
	#32	5’-GCTCTTGATATGGGATCTTT-3’
	#33	5’-GTTCCGTTTGAATGTCTTGA-3’
	#34	5’-CGAGTATTCTTAGGTGCAGA-3’
	#35	5’-AGGAATCATGCCGAGTTTTT-3’
	#36	5’-TTGCTTTCAATTGACGTGCT-3’
	#37	5’-ATAGAAATCGTCGTAGCCGT-3’
	#38	5’-AGGTGAATACGTCTGACACC-3’
	#39	5’-TGAAACTCTCACTTCTCGGG-3’
	#40	5’-GCGCCAATGACTCTTTTTAA-3’
	#41	5’-TTCACAATGCGGGTTAATCC-3’
	#42	5’-GGATTTGTTTTAGACCAGCA-3’
	#43	5’-CTTGATTTTTCCTTCCTTTT-3’
	#44	5’-GCATCTGGGGCTTTTATAAT-3’
	#45	5’-TATCAGTTGTCTGCATCACA-3’
	#46	5’-TTTTCTTGCTTTTCGCTTTT-3’
	#47	5’-AGCTTGAACCCAGATATTGT-3’
	#48	5’-TAAATGCCGGGCTTAGTATC-3’

mei4+ mRNA	#1	5’-TTCGACATTCCCTTGATTTT-3’
	#2	5’-AGTATGACCTTTTTGGGTTT-3’
	#3	5’-CTTCAGTTTCGCATTTTTCT-3’
	#4	5’-GGTTTTCTTTATTCTCTTCA-3’
	#5	5’-TGTCGTATACTGTCACTACT-3’
	#6	5’-GCCATTTCATCTCCAAATAT-3’
	#7	5’-TTCACCAGTATCCACAAAGC-3’
	#8	5’-TAGCATAAGAACACGGTGGT-3’
	#9	5’-TAGGATCGCCAAACCGATTA-3’
	#10	5’-GTCAGTTGCTTGTTATGTGA-3’
	#11	5’-AATCCACGTATAAATCCCGC-3’
	#12	5’-ATCATGGTTGAGGTAGTAGC-3’
	#13	5’-GTTGTGCCTAATGCTATTTT-3’
	#14	5’-AAAGTTTTACCCTTGGGTTT-3’
	#15	5’-AAGTTCTGCATATGGTCAGG-3’
	#16	5’-GAACGATGTAAGCGAACGCT-3’
	#17	5’-ACTGTTTGAGTCAGTTGAGT-3’
	#18	5’-ATTTGCTTGAAGGTCGCTTT-3’
	#19	5’-TCGTCAACGGTTTGATTTCA-3’
	#20	5’-CGAGCTAATGGTATCTCTCG-3’
	#21	5’-AGAGTTTAATCGACTTCGCT-3’
	#22	5’-CGAGCCAGAAGTTGATGTTG-3’
	#23	5’-CTTCGGCAGCAACATTTGAA-3’
	#24	5’-TTGATGGTTGACTTGCATCG-3’
	#25	5’-ATTAGAAGGGGGAAGAGGGG-3’
	#26	5’-GGTTTAGGTACATTTTCTGA-3’
	#27	5’-GATCTTCTTGAGTTTCAGCA-3’
	#28	5’-TAAAGGCTAGACTCATGGGC-3’
	#29	5’-GACGAGAATCGTTGACGTTT-3’
	#30	5’-CCCAGTATTTAATGCCATAT-3’
	#31	5’-TAACCAGGATCTGCATCAGA-3’
	#32	5’-CTGTTATGGCTATTAGAGCG-3’
	#33	5’-CTTCATTGGCAGAATACGGA-3’
	#34	5’-AATCTGCCTGTAGAACATCC-3’
	#35	5’-GCATGGAATGAGGATCTCGA-3’
	#36	5’-ATGGGTTCCCTACGATAATA-3’
	#37	5’-AACGGGATGATGGACGTAGT-3’
	#38	5’-GTTGGGCGTGTATATTCATA-3’
	#39	5’-AAGAGGTATCTGTACGACCG-3’
	#40	5’-GTTGAACAAAAAGCGCCGGG-3’
	#41	5’-GGACTTGAAGGTGCACATTT-3’
	#42	5’-CATGTTCGCGATGTTTTTGT-3’
	#43	5’-ATCCATCGAAAGAGGGTGTT-3’
	#44	5’-TGACTTGAAGGATTCCACGG-3’
	#45	5’-CAAACAAAGGCTCCGAGAGC-3’
	#46	5’-GTCCCTAAAACATGTTACCG-3’
	#47	5’-TAGCCACTTGACTCATGATT-3’
	#48	5’-ACGCTCGATTAGAAGGCATT-3’
