# Supplementary material for: A scaffold lncRNA shapes the mitosis to meiosis switch
Source: Nat Commun. 2021 Feb 3;12:770. doi: 10.1038/s41467-021-21032-7 (PMC7859202; doi:10.1038/s41467-021-21032-7)
Supplement: Supplementary file 2 — Description of Additional Supplementary Files [file 41467_2021_21032_MOESM2_ESM.pdf]

Description of Additional Supplementary Files

Supplementary Data 1 (related to Fig. 2): Analysis of sequential RIP-sequencing (seqRIP-seq) data.
Shown are the features reproducibly enriched upon sequential immunoprecipitations of Mmi1 and Mei2 from biological duplicates.

Supplementary Data 2 (related to Fig. 3): Analysis of RNA-sequencing data.
Shown are the up-regulated and down-regulated genes in *mot2*Δ, *mamRNA*Δ and *mot2*Δ *mamRNA*Δ cells relative to the *wt* strain, and the associated *p*-values.
